# How Do General Practitioners (GPs) Engage in Falls Prevention With Older People? A Pilot Survey of GPs in NHS England Suggests a Gap in Routine Practice to Address Falls Prevention

**DOI:** 10.3389/fpubh.2019.00032

**Published:** 2019-03-11

**Authors:** Lynette Mackenzie, Anne McIntyre

**Affiliations:** ^1^Faculty of Health Sciences, University of Sydney, Sydney, NSW, Australia; ^2^Occupational Therapy, College of Health and Life Sciences, Brunel University London, Uxbridge, United Kingdom

**Keywords:** general practice, allied health practitioners, accidental falls, aging, primary health

## Abstract

Falls are highly prevalent amongst older people and have substantial financial and social costs for health services and the community. Prevention of falls is the key to managing this threat to older people. General practitioners can identify older people at risk of falls on their caseloads. Once identified, actions can be taken to reduce the risk of falls by referring to appropriate services available in the community, such as allied health practitioners. However, the level of engagement in evidence based falls prevention by GPs is unknown. This study aimed to explore how British general practitioners (GPs) address falls prevention with older people, and to determine if there are any gaps in practice. As a pilot study, another aim was to test the feasibility of methods to survey GPs, if a larger survey was warranted from the findings. An on-line cross-sectional survey was distributed by email to all the Clinical Commissioning Groups in NHS England (*n* = 213) and individual general practices listed on the NHS Choices website, supplemented by invitations distributed to CCGs through Twitter and LinkedIn sites. Thirty-seven responses were received. Most GPs were unfamiliar with the 2013 NICE guidelines on assessment and prevention of falls in older people (51.4%, *n* = 19), and only 29.7% (*n* = 11) asked older people if they had fallen during consultations. If falls risk was identified, 81.1% (*n* = 30) frequently made referrals to physiotherapy (PT) and 56.8% (*n* = 21) to occupational therapy (OT). Most GPs did not identify older people on their caseloads as being at risk of falls unless they presented with a fall, and referral rates to relevant AHPs or falls prevention programs were low. Barriers to implementation of falls prevention best practice were identified. Alternative methods are needed to capture the falls prevention practice of a wider sample of GPs.

## Introduction

The high incidence of falls and fall injuries in older people living in the community, combined with the aging population, makes falls prevention a high priority for healthcare professionals. In this paper, the following definition of a fall will be used: “an unexpected event in which the participants come to rest on the ground, floor, or lower level” Lamb et al. [(1), p.1619]. In the UK, older people aged 65 and over are at highest risk of falling, with 30% of people over 65 years and 50% of people over 80 years falling at least annually (2). Injury from a fall is the most common cause of emergency hospital admissions for older people, and around 40% of ambulance attendance is related to older people (3). One study identified that the total cost to the UK government from hospital admissions due to unintentional falls was almost £1 billion, with over half of this cost incurred by the National Health Service and the remainder by the Personal Social Services for long term care (4). Hip fractures are serious injuries resulting from a fall. A significant increase in hip fractures in the UK in the last two decades suggests that if these rates continue to rise, hip fractures following falls could account for up to 140,000 hospital admissions each year, by 2036 (5). Currently, the overall costs of falls to UK healthcare services are estimated at £2 billion a year (6). Monetary costs aside, falls significantly impact on disability outcomes for older people, can be a major precipitating factor for older people moving from their own home into long-term nursing or residential care (7), and are the leading cause of death for older people (8).

Falls risk factors have been identified over several decades of study since the early 1980s. These risk factors are commonly divided into intrinsic risk factors (those that are related to the health of the person e.g., arthritis, vision), extrinsic risk factors (those that are related to the environmental characteristics of a person e.g., home hazards) and behavioral risk factors (individual cognition, insight, attitudes, and distraction e.g., decision-making, habits, using ladders, impulsivity) (3, 9). Deandrea et al. (10) undertook a meta-analysis of 74 prospective studies and considered 31 risk factors. The strongest associations identified were history of falls (OR = 2.8 for all fallers; OR = 3.5 for recurrent fallers), gait problems (OR = 2.1; 2.2), walking aids use (OR = 2.2; 3.1), vertigo (OR = 1.8; 2.3), Parkinson disease (OR = 2.7; 2.8), and antiepileptic drug use (OR = 1.9; 2.7). Other risk factors were identified but could not be evaluated due to a variety of definitions and measurement techniques used in the studies selected for the review. Some risk factors for falls are modifiable such as gait and mobility problems, visual deficits, environmental hazards, medication management and incontinence (9), however, other risk factors are not (such as age and gender).

Falls prevention strategies have the potential to reduce falls and falls injuries by between 10% (11) and 40% (12). Despite evidence that some falls prevention interventions are effective (12), falls rates and rates of hospitalizations due to a fall continue to increase [(13), p.45]. The latest Cochrane systematic review of community based fall interventions (12) and other systematic reviews and meta analyses (14, 15) demonstrate continued support for balance and strength exercises, home safety interventions and medication reviews as effective in preventing falls in community residing older people. A meta-analysis of exercise interventions for fall prevention (15) found that exercise reduced the risk of falling by 17% (RR = 0.83, 95% CI 0.75–0.91). This review provides a pooled estimate across 44 exercise trials. Results also indicated that best effects were observed when high doses of exercise (>50 h) were included in a program, when challenging balance exercises were included and excluded walking programs. A meta-analysis of randomized trials of environmental interventions (14) found a significant reduction in the risk of falling of 21% for all six trials (*n* = 3,298) RR = 0.79 (CI: 65–0.97). Highest effects were with a sub group of people at high risk of falls with existing risk factors such a poor vision (RR = 0.61 CI:0.47–0.79), although protective effects were also achieved for older people at lower risk of falls. Pit et al. (16) also conducted a cluster randomized controlled trial which demonstrated the value of medication reviews and academic detailing in reducing falls at 12 months follow up.

The first step in addressing the falls prevention needs of older people is to identify those who may be at risk of falling. Clinical guidelines and other recommendations indicate that older people should be asked about any falls experienced in the previous year and, if relevant, the frequency, context and characteristics of those falls, whenever they are in contact with a health professional (2, 6, 17). Therefore, if general practitioners (GPs) and allied health professionals (AHPs) are to promote the well-being of older people in the community, they will have a key role of identifying older people at risk and linking them with appropriate services to provide evidence based interventions. Primary health care is the most appropriate service delivery model for an effective falls prevention strategy as virtually all older people living in the community will be connected to a GP. Falls prevention in this context will require effective engagement from GPs in liaison with AHPs (particularly occupational therapists and physiotherapists) working in the community. Currently, the level of falls prevention engagement by GPs is unknown. In the UK, GPs are best placed to identify early falls risk in their older patients as they are primary referral agents and gatekeepers for other community services (6), and they have prolonged contact with their older patients. However, GPs tend to work in response to problems that patients present with rather than work preventively, despite this being a key function of primary care services (18). Amongst the older population attending general practice, the risk of falls has been identified in 50% of patients (19). A study of implementation of health assessments and increased screening in general practice boosted the identification of fallers 3-fold (20), supporting the need for early identification of falls risk in the primary care setting. Falls can be prevented. However, few older people are asked by their general practitioner (GP) about falls or are offered interventions to prevent falls (21). Among those GPs that do address falls, few base their falls prevention practice on recognized clinical guidelines (22). No clear model currently exists for engaging GPs in falls prevention, resulting in missed opportunities to fully address the needs of older people in the community (23).

There is limited published evidence about the current practice of British GPs in relation to falls prevention. A small Australian survey study (24) showed that despite identifying previous falls as an important falls risk factor, only 27% of GPs in the study asked about falls during a consultation with an older person. GP referrals to allied health services were low, despite evidence of the effectiveness of AHP falls prevention interventions (14, 15). American studies indicated that GPs identified multiple medications as an important falls risk factor, especially sedatives, antipsychotics and anti-hypertensives, alongside poor mobility, lack of exercise and home hazards. Common barriers for GPs to undertake falls prevention, included needing to prioritize more acute needs or comorbidities of their older patients, lack of time for thorough falls risk assessment and patients not being prepared to address their falls risk (22, 25, 26). One Australian qualitative study reported that GPs usually dealt with falls prevention after an older person presented with a fall, or if the older patient themselves raised concerns about falls, but primary prevention was not usually carried out (27). As the health systems within which GPs work vary between countries, some of the US and Australian findings may not be easily transferable and inform the British context.

Changes to the GP contract in the UK will require GPs to have knowledge and skills in falls prevention, and use referral pathways to involve AHPs as part of GP co-ordination of care packages for those people over the age of 65 (28, 29). All older people will have a dedicated GP personally accountable for their care, and GPs will oversee personalized care plans integrating all services for those that are deemed to have moderate or severe frailty. GPs will need to identify older people on their caseloads to identify levels of frailty. This will help to reduce inequalities in care, improve access to care and enable early, proactive targeted and appropriate interventions for older people in need. The program will focus on the key evidence-based interventions of falls risk identification and annual medication review. As the level of knowledge and skills that GPs have in fall prevention is unknown, this pilot survey research project was undertaken as a first step to investigate how British GPs identify older people at risk of falls. The aims of the study were to determine the current practice of GPs in relation to falls prevention, to explore how GPs identify older people at risk of falls and their understanding of effective falls prevention interventions, to identify the referral practices of GPs to allied health falls prevention services. We also aimed to test the feasibility of the online survey method to gain information about the practice of GPs in fall prevention.

## Materials and Methods

A cross-sectional survey method was used to gather information from practicing GPs. As GPs in NHS England are subject to specific GP contract arrangements, and the GP system operates differently in Scotland, Wales and Northern Ireland, the survey target group was GPs within NHS England.

### Data Collection

An electronic survey using the Survey Monkey™ platform was developed including items based on literature related to GP practice in falls prevention, current British falls prevention clinical guidelines, and results from Australian and US studies. Surveys are an appropriate method to examine health professionals' perceptions and practices, identify educational requirements and inform health services planning and development (30, 31). Survey topics included the perceptions, knowledge and routine practice of GPs in relation to identifying, screening and assessing falls risks in their patients, their falls management and referral practices, and barriers and facilitators to them effectively preventing falls in their older patients. The survey was pre-tested to ensure clarity of survey layout, question design and to confirm survey completion time (32). Only minor changes were required as the survey was adapted from a previous one used with Australian GPs (24). Table 1 provides a full list of survey questions.

**Table 1:** Survey items.

**GENERAL PRACTITIONER AND PRACTICE CHARACTERISTICS**
• Number of GPs in the practice (FT or PT). *Open response*
• Employment of a practice nurse. *Closed response*
• Role of the practice nurse if employed. *Open text response*
• Gender of GP. *Closed response*
• Time practicing as a GP in the UK. *Open response*
• CCG practice is associated with. *Open response*
• Estimation of total caseload number. *Open response*
• Estimation of % of caseload over 65 years. *Open response*
• Estimation of number of people over 65 years seen per week. *Open response*
• Estimation of the number of people over 65 years seen each week with a risk of falls. *Open response*
**FALLS PREVENTION PRACTICE REGARDING OLDER (65+ YEARS) COMMUNITY DWELLER**
• Routine discussion of falls history with older people aged 65+ during a consultation. *Closed response*
• Routine discussion of fear of falling with older people aged 65+ during a consultation. *Closed response*
• Familiarity with recommendations for screening for falls risk in the 2013 NICE guidelines on the Assessment and Prevention of Falls in Older People. *Closed response*
• Actions taken as part of usual practice in the last year to screen for falls risk in older people aged 65+ in the past year. *Closed response*
• Frequency older people should be screened for falls. *Closed response*
• Most important fall-risk factors for GPs to address in their routine practice (listed risk factors) *Closed response*
• Key barriers preventing GPs from providing falls risk screening as part of routine practice. *Closed response*
• First step taken when a patient is identified with a falls risk. *Closed response*
• Falls assessments that should primarily be conducted by a GP or delegated to a Practice Nurse or appropriate Allied Health Professional (AHP) or medical specialist. *Closed response*
• Identification of local Allied Health Professionals (AHPs) or other health care providers (NHS or private) for falls prevention assessment or interventions. *Closed response*
• Necessary AHPs to provide evidence-based falls prevention interventions. *Closed response*
**COMMUNITY FALLS PROGRAMS AND ALLIED HEALTH PROFESSIONALS (AHP's)**
• Estimate of number of referrals made per year to any AHPs related to FALLS RISK INTERVENTIONS for people aged 65+ living in the community. *Open response*
• AHPs referred to for FALLS PREVENTION interventions for older people living at home. *Closed response*
• Other community-based fall prevention services referred to. *Open text response*
• Most important barriers to referring to FALLS PREVENTION interventions more often. *Closed response*
• Further comments about Falls Prevention in General Practice. *Open text response*

*All items excluded nursing home residents*.

*Close responses included a list of options and an “other” category with a comments box for open text comments if required*.

*Open responses requested numbers*.

*Open text responses invited qualitative comments*.

Ethical approval was granted by the University of Sydney, Australia (approval no: 2013/610) and Brunel University, London, UK. As the survey was focused on service evaluation, did not involve the randomization of participants, did not involve changing patient care and did not anticipate generalizable findings, it was not considered research that needed NHS ethical approval (33). All practicing GPs in NHS England were eligible to participate. All clinical commissioning Groups (CCGs) listed on the NHS Choices website at the time of the survey (*n* = 212) were sent an email with a link to the survey, and asked to distribute the link to their individual GPs to complete. This was supplemented by invitations with the survey link sent out to CCGs through Twitter and LinkedIn sites. Additionally, 379 emails were sent to GP surgeries where email addresses were published on the NHS Choices website. Of these, 66 were returned as undeliverable. A reminder was sent out to all the email addresses 3 weeks after the initial invitation.

### Data Analysis

All returned surveys could not identify participants. Responses were checked, coded and manually entered into SPSS for analysis. Descriptive statistics were used for initial analysis. Relationships between GP characteristics and clinical practices were identified. Any open-text written answers to the questions identified in Table 1 were collated. These were independently coded by the researchers and then reviewed for consensus. Themes emerging from the codes were discussed by both researchers and were related to GP behavior and practice in relation to falls risk screening and assessment, and the key issues identified by participants.

## Results

### General Practitioner and Practice Characteristics

**Figure 1:**
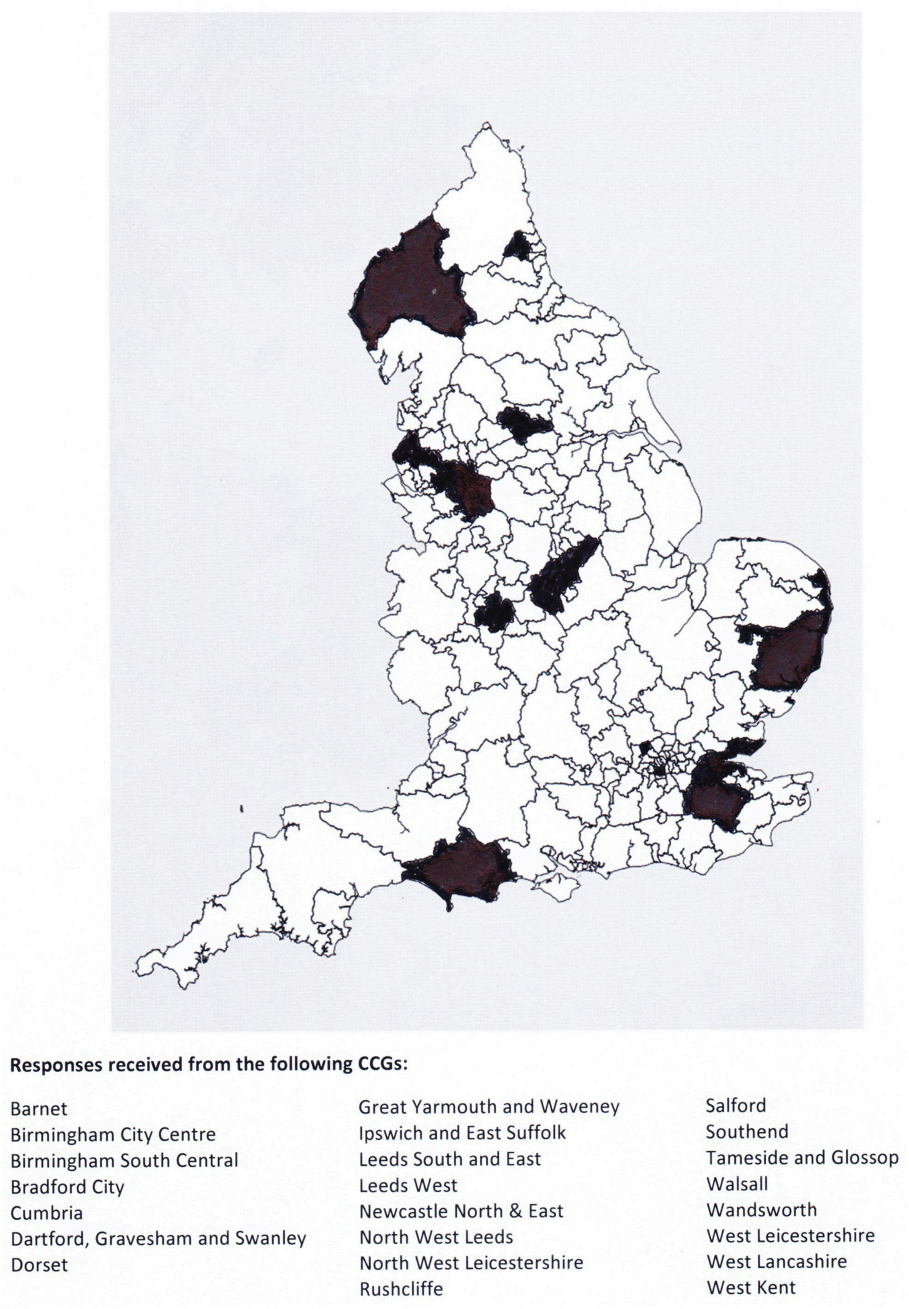
CCG areas that responded to the survey.

Only 37 responses were received to the survey. Because of the multiple methods used to distribute the survey, and the reliance on CCGs to distribute the survey link, it is not possible to calculate an accurate response rate. However, responses were received from 8% of the CCGs. The minimum number of GPs who may have been emailed the request was 591 (212 CCGs + 379 additional GPs), providing a response rate of 6%. The responses were from across the NHS England catchment area (see Figure 1). The GPs who responded from the 22 CCGs indicated in Figure 1 demonstrate that responses were received from GPs in urban, rural and regional areas of NHS England.

Most GP respondents were female (*n* = 26, 70.3%), worked in practices of 0–5 GPs (*n* = 17, 46%), and 97% employed a practice nurse. Compared to available data on GPs in NHS England, this sample was underrepresented by male GPs but the range of GP practice size was broadly representative. Most GPs had practiced in the UK for more than 20 years (*n* = 13, 35%). Respondents estimated that a quarter of their caseload consisted of people over the age of 65 years and approximately half of the older people seen each week were at risk of falls (see Table 2).

**Table 2:** Demographics and practice characteristics of survey respondents (*n* = 37).

	***n***	**%**	**NHS%**
**NUMBER OF GPs IN THE PRACTICE**			
0–5	17	45.9	51.2^*^
6–10	16	43.2	39.4^*^
11+	4	10.9	7.3^*^
**PRACTICE EMPLOYS A PRACTICE NURSE**			
Yes	36	97.3	88.9^**^
No	1	2.7	11.1^**^
**GENDER OF GP**			
Male	11	29.7	45.8^*^
Female	26	70.3	54.2^*^
**LENGTH OF TIME PRACTICING AS A GP IN THE UK**			
0–5 years	5	13.5	
6–10 years	4	10.8	
11–15 years	9	24.3	
16–20 years	6	16.2	
20+ years	13	35.1	
	Mean	95% CI	
Estimated % of people over 65 years on caseload (excluding residential care)	25.9	16.42–35.39	27.4%^***^
Estimated numbers of people over 65 years seen each week (excluding residential care)	55.43	27.54–83.32	
Estimated numbers of people seen each week over 65 years considered at risk of falls (excluding residential care)	22.19	0.00–44.38	

* Data available from http://www.content.digital.nhs.uk/catalogue/PUB24053

** Data available from https://digital.nhs.uk/data-and-information/publications/statistical/general-and-personal-medical-services/final-31-march-and-provisional-30-june-2018-experimental-statistics

*** Data available from https://fingertips.phe.org.uk/profile/general-practice/data#page/11/gid/2000005/pat/152/par/E38000001/ati/7/are/B83620

### Falls Prevention Practice Regarding Older (65+ Years) Community Dwellers

Routine screening practices by GPs for falls risk with older patients were infrequent, according to survey respondents. A third of GPs routinely asked older people during a consultation, if they had previously had a fall, despite over 80% of them identifying previous falls as an important falls risk factor. Only a quarter of respondents indicated that they were familiar with the NICE guidelines for falls prevention (2) and implemented them in their practice. Very few GPs indicated that they routinely used any accepted falls prevention screening practices. For instance, the Timed Up and Go test (34) is a commonly used, simple method to identify falls risk which can be conducted easily. Medication-related screening practices were also not undertaken routinely by most respondents with only up to 60% of GPs identifying falls risk associated with medications. These findings were inconsistent with the high proportion of GPs indicating that mobility and gait issues, and medication issues were important to address. Whilst most GPs indicated that medical, medication and mobility-related falls risk factors were most important for them to attend to, less than half of respondents identified other known risk factors for falls such as home hazards, urinary incontinence and depression (see Table 3).

**Table 3:** Falls prevention practice and opinions of GPs (*N* = 37).

	***n***	**%**
**ROUTINELY ASKS OLDER PEOPLE ABOUT FALLS DURING A CONSULTATION**		
Yes	11	29.7
No	24	64.9
**ROUTINELY ASKS OLDER PEOPLE ABOUT FEAR OF FALLS DURING A CONSULTATION**		
Yes	7	18.9
No	28	75.7
**FAMILIARITY WITH THE 2013 NICE GUIDELINES ON FALLS PREVENTION**		
Familiar and implements them in practice	9	24.3
Familiar but doesn't utilize them	7	18.9
Unfamiliar	19	51.5
**ROUTINE SCREENING PRACTICES TO IDENTIFY RISK OF FALLS**		
Conduct a Timed Up and Go test	4	10.8
Conduct a Sit to Stand test	7	18.9
Conduct an Alternate Step test	2	5.4
Identify patients on 4 or more medications	19	51.5
Identify patients on anti-psychotics	14	37.8
Identify patients on anti-hypertensives	20	54.1
Identify patients on sedatives	21	56.8
**MOST IMPORTANT FALLS RISKS GPs SHOULD ATTEND TO**		
Postural hypotension	32	86.5
Multiple medications	31	83.8
Past falls history	30	81.1
Gait and balance problems	29	78.4
Psycho-active medications	28	75.7
Dizziness	28	75.7
Frequent slips and trip	27	72.9
Use of a mobility aid	26	70.3
Parkinson's Disease	24	64.8
Alcohol or drug abuse	22	59.5
Poor vision	21	56.8
Peripheral neuropathy	20	54.1
Syncope	20	54.1
Sedentary lifestyle/lack of exercise	18	48.6
Home hazards	17	45.9
Depression/anxiety	17	45.9
Inadequate nutrition	17	45.9
Arthritis	17	45.9
Osteoporosis	17	45.9
Foot pain or poor footwear	16	43.2
Muscle weakness	13	35.1
Urinary incontinence	13	35.1
Poor sleep	13	35.1
Diabetes	12	32.4
Vitamin D deficiency	12	32.4

When asked how often an older person should be screened for falls risk, respondents indicated only when a patient reported falling (*n* = 27, 77.1%), during a general health check (*n* = 23, 65.7%), during other related consultations (*n* = 22, 62.9%), once a year (*n* = 10, 28.6%) or as part of every consultation (*n* = 3, 8.6%). A total of 64 qualitative comments were made by respondents in free text boxes associated with each question in the survey. Two key themes emerged from the qualitative comments. First, some GPs acknowledged their own lack of attention to falls in their practice with older people and therefore, their associated lack of knowledge about falls, or where to refer to when seeking assistance. For one participant, this was related to system barriers. For instance:

“*Ideally [falls screening should happen] every consultation BUT in 10 minutes that is not possible unless it is the reason for the patient's consultation.”* (P7. Female, 3 GPs in practice, worked in the UK for 20+ years).

For another participant, this was attributed to a lack of awareness. For instance:

“*Doesn't cross my mind enough, and sometimes not quite sure who to refer to.”* (P.19. Female, 3 GPs in practice, worked in the UK for 0–5 years).

Secondly, other GPs indicated that they would independently manage their clients and would trust their own judgement about when falls screening might be needed. For instance:

“*I don't routinely screen for falls risk. This is not part of my usual practice in any defined way.”* (P.37.Female, 3 GPs in practice, worked in the UK for 0–5 years), and:

“*I mainly try to sort out the patient myself.”* (P17. Male, 9 GPs in practice, worked in the UK for 11–15 years).

### Barriers to Falls Prevention in GP Practice

Barriers identified by GPs to undertaking falls risk screening and referring to AHPs predominantly related to lack of time and a busy, complex caseload (see Table 4). Smaller proportions of respondents expressed doubt about the effectiveness of falls prevention interventions, and whether patients would willingly engage in falls prevention activities.

**Table 4:** Barriers to falls prevention for GPs (*n* = 37).

	***n***	**%**
**BARRIERS IDENTIFIED TO FALLS PREVENTION SCREENING BY GPs**		
Time restraints in clinical practice	33	89.1
Patients have more immediate demands when they attend a consultation	30	81.1
Priorities and trade-offs with competing comorbidities	21	56.8
Issue too complex for one surgery visit	13	35.1
Patient denial they are at risk	12	32.4
Seeing the same patient infrequently	5	13.5
No incentives to do this	4	10.8
**BARRIERS TO REFERRAL TO ALLIED HEALTH PROFESSIONALS FOR FALLS PREVENTION SERVICES**		
Time restraints	19	51.4
Patients would be unwilling to be referred	13	35.1
Busy workload	12	32.4
Lack of local NHS AHPs	7	18.9
Don't know who to refer to	4	10.8
Don't believe a referral would be effective	3	8.1

### Assessment of Falls Risk Factors in GP Practice

**Figure 2:**
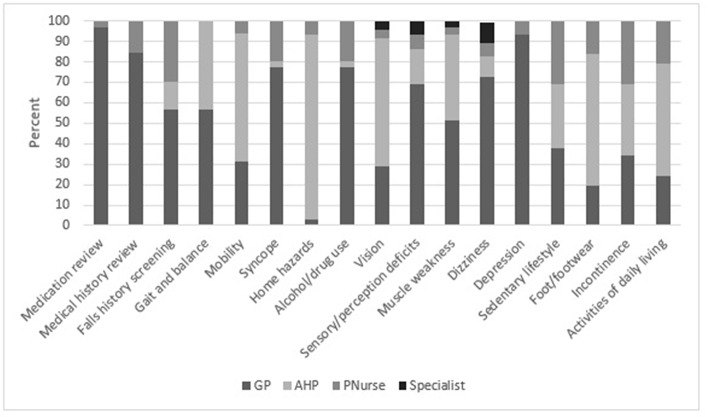
GP opinions on who should asses each falls risk factor in older people (*N* = 30).

Assessment of activities of daily living, foot pain, footwear, mobility and vision were considered as the primary role of the GP by 20–30% of respondents. However, most GPs indicated they would delegate some assessments to AHPs such as gait, mobility, home hazards, vision, muscle weakness, sedentary lifestyle, foot pain or footwear, incontinence and activities of daily living (see Figure 2).

### Community Falls Programs and Allied Health Professionals (AHP's)

GPs indicated that they made an annual mean number of 22.8 (95% CI 11.8–33.8) referrals to any AHP in a year. If GPs identified a patient with falls risk, they indicated that they would initially refer to a NHS falls clinic (*n* = 15, 42.9%), undertake a more in depth medical assessment appointment themselves (*n* = 13, 37.1%), directly refer to an AHP (*n* = 6, 17.1%) or refer to a geriatrician or other specialist (*n* = 1, 2.9%).

## Discussion

This study aimed to explore the current practice of British GPs in relation to falls prevention, to determine if there was a gap in practice, and to test the feasibility of survey methods to gain this information. Findings indicate a lack of awareness of clinical guidelines for falls prevention services by these GPs, with most not pro-actively identifying older people on their caseloads as being at risk of falls, and low referral rates to AHPs or falls prevention programs. Very few GPs routinely asked older people if they had experienced a fall during consultations, and qualitative data indicated that GPs in this small sample often overlooked investigating falls or identifying older people on their caseload with an increased risk of falls. It appears that respondents did not think about or engage in falls prevention screening, irrespective of their length of time in practice, but why this was the case is not known from the survey responses. This was despite GPs estimating that a mean number of 22 older people with falls risk factors were seen by them each week (Table 2).

There were inconsistencies about ratings given by GPs about what they thought was important to prevent falls, and how they implemented these into their practice. For instance, although previous falls and gait problems were rated as important, GPs did not ask about falls, or undertake simple tests of gait during their consultations with older people. Despite citing lack of time as a serious barrier to undertaking falls prevention activities, many GPs indicated that they should be the primary health professional that assessed many falls risk factors rather than delegating this to other health professionals who were specialized in the area. Clearly there is a gap in routine falls prevention practice which should be investigated more comprehensively beyond the limitations of this pilot study. Furthermore, whilst high numbers of respondents indicated that some AHP's were important to refer to, this was not matched by the frequency of reported referrals to the same AHP groups (see Table 5).

**Table 5:** GP referral practices and opinions about AHPs and falls prevention (*N* = 37).

	***n***	**%**
**AHPs NECESSARY TO PROVIDE EVIDENCE BASED FALLS PREVENTION INTERVENTIONS**		
Physiotherapy	32	86.5
Occupational therapy	31	83.8
Optometrist/orthoptist	21	56.8
Pharmacist	17	45.9
Podiatrist	16	43.2
Social worker	14	37.8
Registered nurse	9	24.3
Dietitian	8	21.6
Exercise physiologist	7	18.9
Psychologist	6	16.2
Complimentary medicine (acupuncture etc.)	2	5.4
**AHPs THAT GPs MOST FREQUENTLY MAKE REFERRALS TO FOR FALLS PREVENTION INTERVENTIONS**		
Physiotherapy	30	81.1
Occupational therapy	21	56.8
Falls clinic	16	43.2
Social worker	5	13.5
Exercise classes	4	10.8
Podiatrist	3	8.1
Optometrist/orthoptist	2	5.4

In relation to feasibility findings, the low response rate may indicate that the use of online surveys is not likely to encourage responses from GPs, who may receive multiple competing emails for their attention. From a research perspective online surveys are a very efficient and economical way to gather and download data, however, this benefit may be lost if GPs are less likely to respond to an online survey rather than a hardcopy survey. For those that did respond the survey did elicit important information about the fall prevention practice of GPs, suggesting that the survey itself was designed well. More comprehensive responses may have been gained by involving CCGs in the early stages of study design for them to actively support dissemination and response to the online survey (35).

These findings are consistent with studies from the US and Australia, indicating that few older people are asked by their GP about falls or are offered interventions to prevent falls (21, 24), and few GPs practice according to recognized clinical guidelines (22, 24). Clinical guidelines recommend that in the first instance older people should be regularly asked about any falls (2, 6, 17, 36). Guidelines also recommend specific interventions that older people should be given to reduce their risk of falls (2, 37, 38). These interventions include minimizing medications, tailored exercise programs, treating vision impairment, managing postural hypotension, managing heart rate and rhythm irregularities, vitamin D supplementation, managing foot and footwear problems, modifying the environment and providing education. Most of these interventions will require GP referral to access services in the community.

Several barriers to providing evidence-based falls management were reported, which are also reflected across the literature. A slow take up of allied health services to address falls that has also been attributed to organizational barriers, difficulty for GPs in initiating the process (39) and a lack of understanding of what AHPs can offer (40). This survey indicates that little appears to have changed since these studies were published. The barriers to implementing evidence based falls prevention practice expressed by survey respondents are confirmed in the literature (41). These include practical issues such as access and time, appreciating relevant social and cultural factors, and client preferences about how advice is delivered and by whom. Another systematic review identified that additional training can improve implementation of falls prevention interventions, although this may not be as effective for the management of falls in primary care settings (42). Support is needed to develop accessible pathways to identify older people at risk and to develop better partnerships and collaborations with other health professionals (23). These issues were confirmed by British GPs in these study findings.

### Strengths and Limitations of the Study

This appears to be the first study to identify GP practice in falls prevention in the UK. Study results have provided some preliminary information about the current level of falls prevention activities undertaken in general practice, and have identified that further investigations are needed to facilitate better falls prevention practice at the primary care level. The low response rate is a major limitation and results should be interpreted with caution as they cannot be generalized. However, the initial findings are important enough to instigate further investigation to ensure falls prevention is effectively undertaken in general practice once the gaps in service are known. It is well documented that there are difficulties in gaining adequate response rates from GPs for survey research (43–45), and GP response rates in published studies can be under 30% (43), or as low as 0.1% (46). For this study, the use of on-line surveys and invitations may have contributed to the low response rate (47), and as a non-funded study, there were no funds to offer monetary incentives to GPs to participate. Referring to postal surveys, Edwards et al. (48) identified the following techniques known to increase survey responses: monetary incentives, short surveys, personalized questionnaires and letters, use of colored ink, use of stamped return envelopes, contacting participants before sending surveys, follow up contact and providing non-respondents with a second copy of the survey. When compared with online surveys used with GPs, the participation rate in online surveys has been shown to be much lower than those delivered by mail (49), although mailed surveys also have consistently reported low response rates. Therefore, despite the benefits of using online surveys as was done in this pilot study, future studies should also use postal surveys to maximize responses. Future surveys will need to be designed as short as possible, be offered online and by mail with stamped return envelopes, and include an incentive to participate. Alternatively, strategies such as telephone or even face-to-face interviews with GPs may be more successful. Collecting data at conferences attended by GPs and supporting the survey by advertisements in GP journals are other possibilities. However, these strategies are also costly in terms of resources and time.

### Implications for Research and Practice

Falls experienced by older people in the community are preventable, but only if older people at risk of falls are identified and their falls risk is adequately managed. The latest Cochrane systematic review of community based fall interventions (12) demonstrated the effectiveness of balance and strength exercises, home safety interventions and medication reviews for preventing falls in community residing older people. However, the preliminary findings from this study suggest that there are gaps in falls prevention management practice by GPs, and barriers to implementation of falls guidelines in practice. Barriers included lack of time during consultations, other more pressing issues and a lack of educational materials to give to patients (22). More effective partnership between GPs and AHPs is needed. As the majority of GP practices in this study employed a practice nurse, some delegation of falls prevention screening could be possible if practice nurses were offered sufficient training. Education should be offered to GPs, AHPs, practice nurses and patients to ensure that best practice falls prevention is available in primary health settings. Clearly, this study should be replicated with a larger, more comprehensive sample of GPs to confirm the findings, using an alternative form of data collection, and incorporating methods to encourage GP participation.

## Conclusion

Clinical guidelines indicate that older people should be asked regularly about any falls by health professionals but the study findings indicated that this rarely occurs in practice. GPs are in a position to identify older people on their caseload who are at risk of falls and put in place management strategies to reduce this risk at a primary prevention level. However, the findings also describe the primary health context as time poor for GPs. This pilot study justifies a more comprehensive survey across NHS England is needed to confirm these findings, and that paper surveys individually delivered to GPs may be more effective in gaining responses.

## Author Contributions

AM and LM made substantial contributions to the conception and design of the research and the analysis, and interpretation of data for the research, produced drafts of the manuscript and contributed to revising it critically for important intellectual content, approved the final version of the manuscript for publication, agree to be accountable for all aspects of the work in ensuring that questions related to the accuracy or integrity of any part of the work are appropriately investigated and resolved. LM conducted the data collection for the project.

### Conflict of Interest Statement

The authors declare that the research was conducted in the absence of any commercial or financial relationships that could be construed as a potential conflict of interest.
